# Urea–Urease pH-Clock-Enabled Time-Lapse Thiol–Acrylate Adhesives: Two-Part Formulations and One-Pot Underwater Activation

**DOI:** 10.1021/acsomega.6c02018

**Published:** 2026-07-04

**Authors:** Fahima Shaon, Anthony Mai, Md Abdullah Al Mahmud, Jorge A. Belgodere, John A. Pojman

**Affiliations:** † Department of Chemistry and the Macromolecular Studies Group, 5779 Louisiana State University, Baton Rouge, Louisiana 70803, United States; ‡ Tulane Medical Center Hematology and Oncology, New Orleans, Louisiana 70112-2600, United States

## Abstract

This work presents time-lapse thiol–acrylate adhesive systems that use a urea–urease pH-clock reaction to enable programmable curing. Two complementary approaches are demonstrated: a two-part adhesive in which enzymatically generated ammonia provides a tunable induction period prior to rapid gelation, and a one-pot, water-activated formulation that could be used for underwater curing. In the two-part system, mixing a thiol component containing watermelon seed powder (WMSP) with an acrylate component containing an aqueous urea solution initiated a gradual pH increase that delayed the thiol-Michael polymerization, allowing programmable working time. This induction time is unlike other two-part systems that begin to react immediately upon mixing. The cured adhesive exhibited a lap shear strength of 7 MPa on polycarbonate. The one-pot formulation, stabilized with monomethyl ether hydroquinone (MEHQ) and phenylphosphonic acid, remained shelf-stable in the absence of water but cured rapidly upon wetting as the water dissolved the urea, which then was converted to ammonia by the WMSP, achieving lap shear strengths of ∼5 MPa. Together, these systems demonstrate the effectiveness of WMSP-derived enzymatic catalysis for achieving temporal control in thiol–acrylate adhesives.

## Graphical abstract

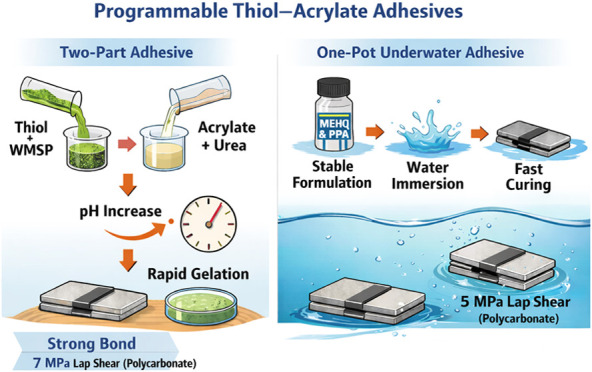

## Introduction

The ability to control the timing of adhesive curing is advantageous in applications ranging from industrial assembly to biomedical sealants.^1–3^ Conventional two-component adhesives (e.g., “5 min” epoxies) begin reacting immediately upon mixing, which fixes a short working time and can be suboptimal when a delayed set or positioning time is needed.^4–7^

An ideal system would remain processable for a programmable period and then rapidly cure. Frontal polymerization, a cure-on-demand technology, has been applied to adhesives,^8–10^ but these systems reach high temperatures that limit their applications to substrates that can tolerate high temperatures.

Thiol–ene reactions are frequently employed in polymer synthesis,^11,12^ as well as in functionalization, surface modification, and hydrogel formation.^13–21^ Thiol–acrylate systems are attractive as adhesive matrices due to their rapid Michael addition reaction under mild conditions, forming highly cross-linked networks without volatile byproducts.^22–27^ The base-catalyzed addition of a multifunctional thiol (R–SH) with a multifunctional acrylate is a step-growth polymerization that proceeds at ambient conditions and is not inhibited by oxygen, unlike free-radical polymerizations.^25,28–30^ These systems can produce thermoset polymers with tunable mechanical properties and strong adhesion without the need for solvents or energy input.^21,22,31^ However, achieving temporal control over thiol–acrylate curing remains challenging, as Michael addition reactions begin to set as soon as the base catalyst is introduced.^24,26,30^

Chatani et al. developed a thiol–ene system with temporal control.^31^ They showed that a combination of an electron-deficient vinylic species, a nucleophile, and an acid can generate predictable induction periods for base-catalyzed thiol-Michael addition reaction. Moreover, the length of the induction time was readily tuned by adjusting the concentrations of each component within the initiating system.

The use of pH-clock reactions to trigger polymerizations has been reported. Such reactions exhibit a large pH change after a programmable “clock time”.^32–34^ The first pH-clock reaction used was a formaldehyde clock reaction to trigger the thiol–acrylate Michael addition.^35^ To avoid the toxicity of formaldehyde, we switched to the urea–urease system. The hydrolysis of urea to produce ammonia and carbon dioxide is catalyzed by urease (Figure 1). It was shown that the production of base and the pH dependence of the urea–urease reaction give rise to base-catalyzed feedback.^36,37^

**Figure 1:**
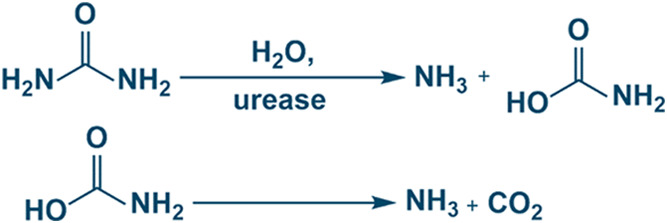
Hydrolysis of urea in the presence of the urease enzyme.

Jee et al. demonstrated a time-lapse polymerization of an aqueous thiol–acrylate solution based on the urea–urease reaction.^38^ They proposed that NH_4_OH produced by the hydrolysis of urea catalyzed the thiol–acrylate reaction. Pojman received a patent on this time-lapse polymerization approach.^39^ Mai et al. demonstrated that an extract from watermelon seeds provides a source of urease, which they called “watermelon seed powder” (WMSP).^40^ They also determined that the WMSP is stable for at least a year at ambient conditions. Bashir et al. used this watermelon seed powder with the urea–urease reaction to control the gelation of poly­(vinyl alcohol) by borate.^41^ Other works have shown applications for the urea–urease reaction.^40,42–44^ Recently, Kürsteiner et al. demonstrated that lignocellulosic materials could be consolidated by using urease from watermelon seeds with urea to synthesize struvite.^45^

Mahmud et al. reported a one-pot formulation for cure-on-demand coatings with urea and WMSPs.^21^ The application of water created a solution of urea that produced ammonia, which diffused into the resin layer and served as a catalyst for the addition of a thiol to an acrylate and for the reaction of a thiol with an epoxy.

In this work, we studied two approaches to creating adhesives driven by the urea–urea reaction. In the first system, WMSP and urea were incorporated into separate components of an adhesive formulation to produce a two-part system that remains dormant until mixing. Upon combination, the urea–urease reaction progressively increased the pH and produced ammonia that can diffuse into the organic phase, thereby catalyzing thiol–acrylate polymerization.^21,46^ We term these “time-lapse adhesives” for their ability to delay cure in a programmable fashion.

In the second system, we created a water-activated one-pot adhesive in which all components (thiol, acrylate, urea, and WMSP urease) were premixed and remained inert until water ingress initiates the urea–urease clock reaction. Key to this approach was suppressing reaction during storage. Mahmud et al.^21^ successfully stabilized thiol–acrylate formulations to achieve a long shelf life using phenylphosphonic acid and MEHQ, following the work of Esfandiari et al.^47^ Following this work, we introduced a small amount of MEHQ as a radical inhibitor to prevent acrylate polymerization, and phenylphosphonic acid as a weak acid to keep the thiol groups protonated and the pH low in the absence of water.^7,47,48^

## Experimental Section

### Materials and Methods

Trimethylolpropane tris­(3-mercaptopropionate) (TMPTMP) and trimethylolpropane triacrylate (TMPTA) were purchased from Sigma–Aldrich. The watermelon seed was purchased from Eden Brothers, and the WMSP was prepared following the procedure of Mai et al.^46^ Urea (ACS grade) was acquired from Fisher Scientific. Phenylphosphonic acid (PPA), MEHQ, and fumed silica (Aerosil 200 and R972) were obtained from Sigma–Aldrich and Evonik, respectively. Bromocresol purple was purchased from MilliporeSigma. Deionized water was used throughout the experiments.

TMPTMP and TMPTA (Figure 2) were used as received. For the two-part adhesive, deionized water and hydrochloric acid were used to prepare an acidic urea solution, and PPA and MEHQ were obtained for use in the one-pot formulation. Fumed silica fillers were incorporated to control the viscosity. Hydrophilic fumed silica (Aerosil 200) and hydrophobic fumed silica (Aerosil R972) were used in the specified ratios. Bromocresol purple (pH indicator) was used to visualize pH changes during the cure.

**Figure 2:**
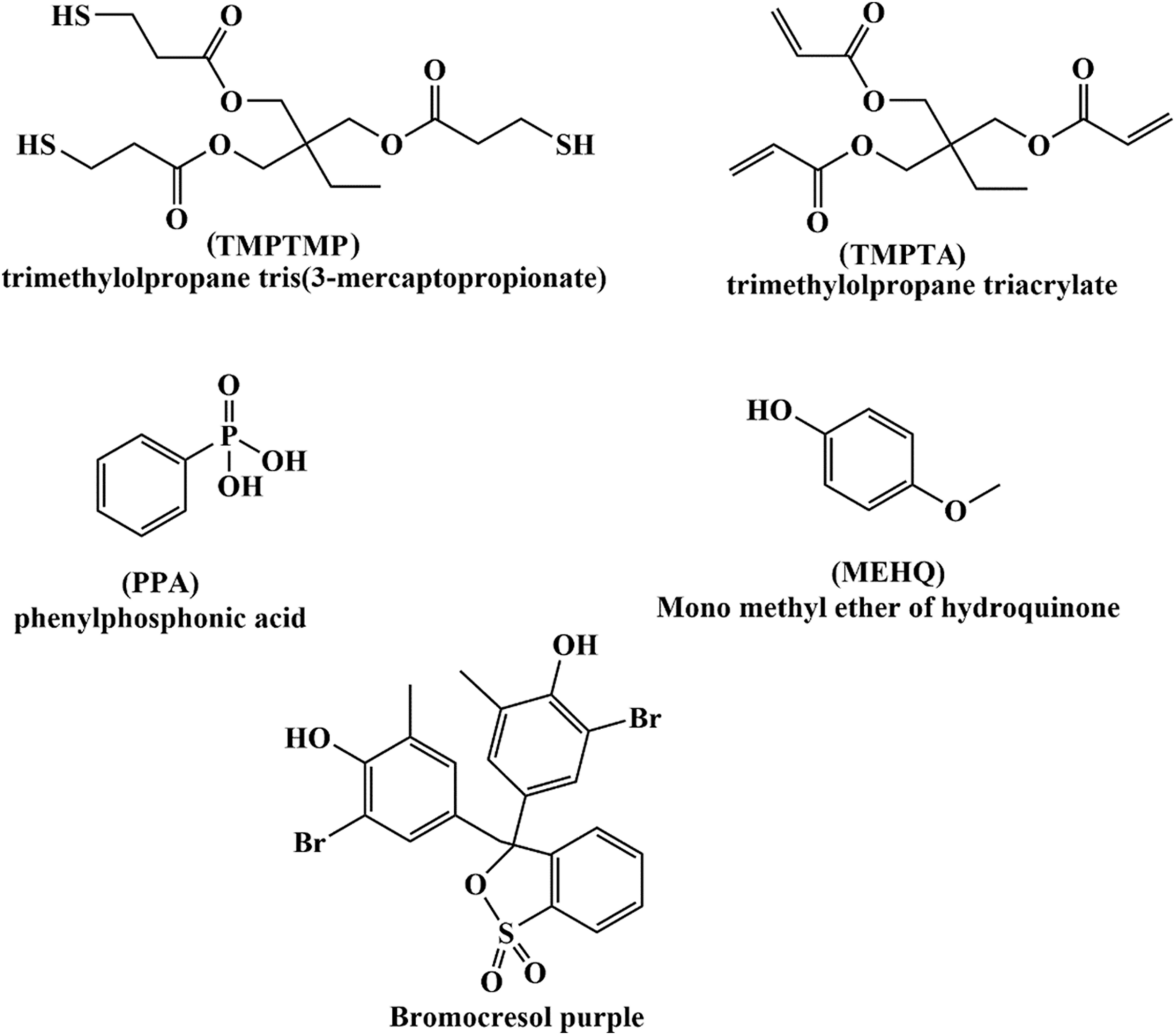
Structures of major chemicals used.

### Two-Part Adhesive Formulation

In the two-part time-lapse adhesive (Table 1), Part A (thiol part) and Part B (acrylate part) were formulated separately and mixed 1:1 (w/w) at the time of use. Part A consisted of TMPTMP (84 wt %), WMSP urease (7 wt %), hydrophilic fumed silica (4.5 wt %), and hydrophobic fumed silica (4.5 wt %). Part B consisted of TMPTA (85 wt %), urea solution (6 wt % of a 30 wt % urea solution in pH 3 water), hydrophilic fumed silica (4.5 wt %), and hydrophobic fumed silica (4.5 wt %). The urea solution for Part B was prepared by dissolving urea in deionized water and adjusting the pH to ∼3 using HCl, creating an acidic aqueous phase (∼5% of Part B by weight) that was dispersed in the acrylate monomer. To prepare each part, monomer and additives were combined and mixed thoroughly until a uniform, paste-like consistency was achieved. The fumed silica served to increase the viscosity and maintain a homogeneous dispersion of the WMSP and the aqueous urea microdroplets, preventing phase separation. Each part was stored in sealed containers at room temperature (or refrigerated for long-term storage studies) until use.

**Table 1:** Optimal Formulation for Two-Part Time-Lapse Adhesive

part A: thiol		part B: acrylate	
materials	amount (wt %)	materials	amount (wt %)
TMPTMP	84	TMPTA	85
WMSP	7	30% urea solution (pH ∼ 3)	6
hydrophilic fumed silica	4.5	hydrophilic fumed silica	4.5
hydrophobic fumed silica	4.5	hydrophobic fumed silica	4.5

The mixing of Part A and Part B started the urea–urease reaction as the WMSP came into contact with the urea solution, generating ammonia. Unless otherwise noted, adhesive samples were prepared by mixing equal masses of Part A and Part B (to form a stoichiometric thiol to acrylate ratio of 1:1 functional groups) and quickly stirring for ∼30 s to ensure uniformity before observing curing behavior or applying to substrates.

The gelation time was determined as the time after mixing, when the formulations could no longer be stirred manually.

### One-Pot Water-Activated Adhesive Formulation

The one-pot adhesive shown in Table 2 was formulated as a single homogeneous mixture containing all components and optimized for stability until water exposure. The optimized formulation included TMPTMP (39 wt %), TMPTA (38 wt %), WMSP (10 wt %), urea powder (8 wt %), hydrophilic fumed silica (2.1 wt %), hydrophobic fumed silica (2.0 wt %), MEHQ inhibitor (0.4 wt %), and PPA (0.5 wt %). To prepare the one-pot adhesive, we first combined the liquid monomers (TMPTMP and TMPTA). Next, MEHQ and PPA were dissolved in the monomer mixture. Then, both the hydrophilic and hydrophobic fumed silica were added incrementally with vigorous stirring to thicken the resin and suspend the powders. Finally, the WMSP and urea powder were added and mixed until a uniform paste was obtained. The resulting formulation was a thixotropic paste in which solid urea and enzyme were distributed. The presence of PPA maintained a low initial pH and kept thiol groups protonated, while MEHQ captured any radicals that could initiate acrylate polymerization.^7,47^ This one-pot adhesive was stored under ambient conditions in sealed containers for stability testing.

**Table 2:** Composition of the Optimized Water-Activated, Time-Lapse Adhesive Formulation Substrates and Surface Preparation

materials	amount (wt %)
TMPTMP	39
TMPTA	38
WMSP	10
urea powder	8
hydrophilic fumed silica	2.1
hydrophobic fumed silica	2
MEHQ	0.4
PPA	0.5

Therefore, to trigger curing, the one-pot adhesive was either mixed with a small amount of water or applied to a substrate and then exposed to water (e.g., submerged or sprayed). Water ingress dissolved urea and PPA, raising the local pH as urease became active and produced ammonia, which in turn catalyzes the thiol–acrylate reaction. In this way, the adhesive can be applied on a wet surface, where it will adhere and cure. For the underwater adhesion tests described below, the one-pot adhesive was spread between substrates, and the assembly was then immersed in water to initiate curing.

The adhesion performance of the developed thiol–acrylate-based time-lapse adhesive was systematically evaluated across various substrates, including polycarbonate, aluminum alloy, balsa wood, glass, and Styrofoam (Figure 3). Prior to adhesive application, surface preparation was performed to optimize adhesive interactions, which involved cleaning with deionized water, followed by acetone. Subsequently, the substrates were dried under ambient conditions to remove residual moisture. Mechanical roughening of metal substrates was implemented via sanding to enhance the surface roughness, thereby promoting mechanical interlocking and significantly increasing the adhesive contact area. The optimal adhesion performance was recorded with polycarbonate, demonstrating the highest adhesive strength. This is surprising, and we do not have an explanation (Figure 3).

**Figure 3:**
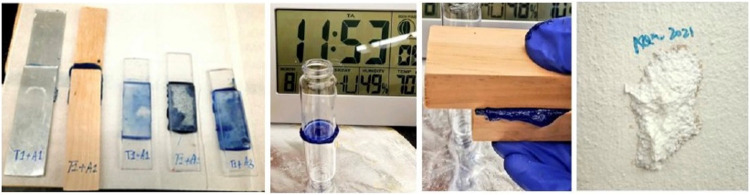
Evaluation of adhesion performance of thiol-Michael addition-based time-lapse adhesives on various substrates, including metal, wood, glass, and wall surfaces.

### Characterization and Testing Methods

#### Lap Shear Adhesion Testing

Adhesive bonding strength was quantified by lap shear tests following ASTM D1002. Substrates of different materials like polycarbonate plastic, aluminum alloy (2024-T3, “metal”), and birch wood were cut into coupons (approximately 100 mm × 25 mm × 2 mm). Prior to bonding, surfaces were prepared by abrading with sandpaper (120 grit) and cleaned with isopropanol. For the two-part adhesive, equal parts of A and B were mixed and applied to an overlap area of 12.5 mm × 25 mm between two coupons (bond line thickness ∼1 mm). The assembly was clamped lightly and left at room temperature for 24 h to ensure full cure. For the one-pot adhesive, the paste was applied to one substrate (dry) at the same bond area, and immediately, a drop of water (∼5% by weight of adhesive) was added and mixed into the adhesive layer with a spatula. The second substrate was then pressed on, and the assembly was either wrapped with a wet paper towel or submerged in water for 1 h to cure. The test was performed on an Instron universal tester with a 50 kN load cell at a shear loading rate of 1 mm/min until failure. The peak load was recorded and converted to lap shear strength (MPa) by dividing by the bonded area. At least five replicates were tested for each condition. After testing, fracture surfaces were examined to identify the failure mode (cohesive vs adhesive).

#### Shelf Life and Pot-Life Evaluation

To assess the shelf life of the two-part system, unmixed Part A and Part B samples were stored at various temperatures (4 °C, ambient ∼23 °C, and 50 °C). At intervals (every 30–60 days), a small portion was taken, mixed, and the gelation time was measured. Lap shear samples were also prepared from aged material to evaluate any decline in cured bond strength over time. For the one-pot adhesive, pot life was gauged by storing the formulation in a sealed syringe at ambient temperature and testing a small, extruded sample for curing ability (by adding water) every week. A successful cure with a similar gel time to fresh adhesive indicated that the formulation was still active.

#### Gravimetric Degradation Study

To study long-term water stability, cured adhesive samples (one-pot formulation cured underwater) were subjected to accelerated degradation. Dumbbell-shaped specimens were molded (25 mm × 4 mm × 2 mm) and fully cured underwater. The samples were then stored in distilled water at 37 °C. At set time points (7, 14, 30, 60, 90 days), specimens were removed, gently patted dry, and weighed to determine mass loss. Lap shear strength tests on these specimens were conducted to gauge retention of mechanical strength over time in water. Any surface erosion or mass loss indicated degradation. After each measurement, samples were returned to fresh water to continue the degradation study.

#### Data Analysis

All numerical data were reported as mean ± standard deviation.

## Results and Discussion

### Enzymatically Programmed Curing in Two-Part Adhesive System

#### Formulation and Working Time

The two-part adhesive was formulated to remain liquid after mixing for a controllable period before rapidly gelling. Upon combining Part A (thiol + WMSP) and Part B (acrylate + urea solution), the mixture initially had a low pH (∼3–4 from the acidic urea solution). This acidic environment suppressed urease activity and kept thiols protonated. During this induction period, the adhesive was flowable and applicable. As the urease in WMSP slowly hydrolyzed urea, ammonia was generated, which gradually raised the pH. After a certain induction time, the pH crossed a threshold that activated the thiol–acrylate reaction. A visible indicator of this process was provided by bromocresol purple: the mixed adhesive initially appeared greenish (indicating pH ∼3–4) and shifted to blue as the pH approached neutral, typically just before gelation (around pH 6–7).

In optimized formulations (with 7–10% WMSP and ∼3–6% urea in the total mixture), the induction times observed were on the order of 5–10 min at room temperature, after which the adhesive rapidly set. The color change from the pH indicator occurred approximately 1–2 min before the gel point, providing a useful visual cue of the impending cure. The induction time and curing profile were tunable by adjusting key parameters. For instance, increasing the urea concentration in the formulation shortened the induction time significantly by providing more substrate for ammonia generation. Raising the urea content from 6 wt % to 12 wt % (in the final mix) reduced the gelation time from ∼60 min to ∼10 min. Likewise, increasing the WMSP (urease) content accelerated curing: with 4–5 wt % WMSP, the mixture remained liquid for over 30 min, whereas with 10 wt % WMSP, the gel time dropped below 5 min.

These trends demonstrated the expected clock reaction behavior—higher enzyme or reactant concentrations produced ammonia faster, thus reaching the critical pH for polymerization sooner. The initial pH of the mixture was another important factor. By formulating the urea solution in Part B at different pH values (from ∼2 to 6), we controlled the initial pH of the mix. A low initial pH (∼2) led to a long induction (>50 min), whereas a slightly higher initial pH (∼5) gave a moderate delay (∼15 min). Interestingly, the ultimate bond strength of the adhesive showed a nonmonotonic dependence on initial pH: lap shear tests revealed a peak lap shear strength around 7–8 MPa for samples cured with an initial pH of 4–6, whereas at pH 2 or pH 8 the strength was lower (typically 4–5 MPa). This indicates that an optimal balance of cure rate and network structure was achieved at an intermediate pH. Also, high pH may cause hydrolysis of the polymer.

#### Mechanical Properties and Adhesion Performance

After curing, the two-part adhesive formed a stiff, opaque polymer matrix (due to dispersed WMSP and silica). Lap shear tests revealed high bonding strength on several substrates (Figure 4). On polycarbonate (PC) plastic sheets, the adhesive achieved an average shear strength of ∼7.1 MPa. The failure of PC was adhesive failure. On aluminum metal substrates, the strength was lower (∼3.3 MPa), and failure was adhesive.

**Figure 4:**
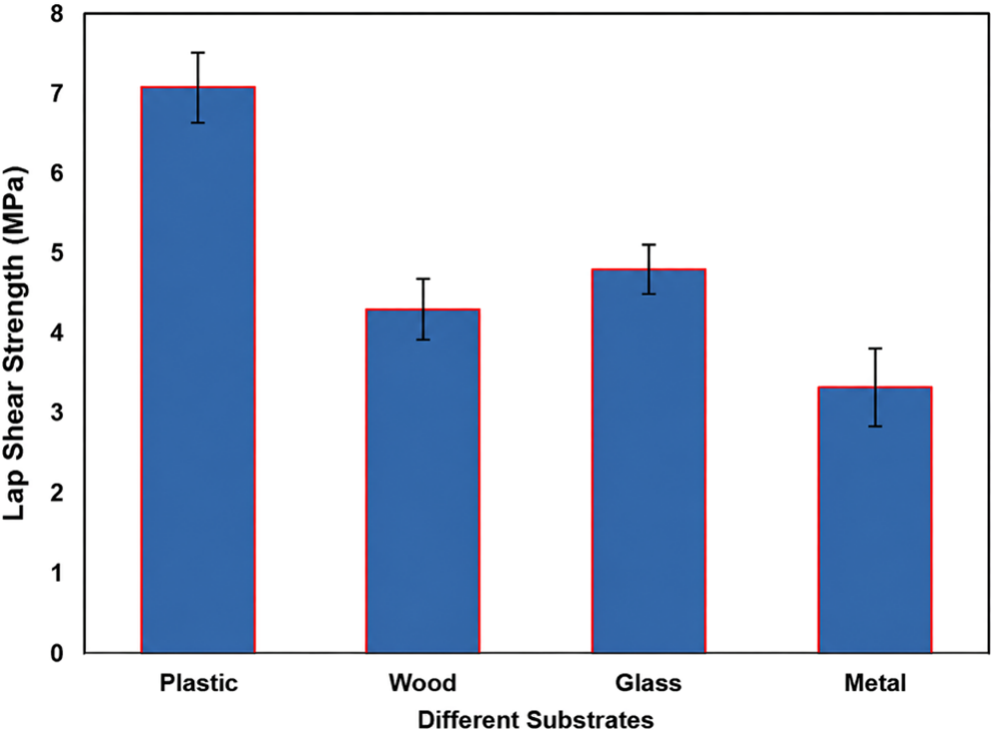
Lap shear strength values of various substrates bonded with the two-part adhesive, evaluating the mechanical performance of the adhesive over time.

This disparity is likely due to poor wetting and interaction of the hydrophobic thiol–acrylate resin with the aluminum oxide layer. On wood (birch) substrates, intermediate performance was observed: ∼5 MPa shear strength, with a mix of adhesive failure and wood substrate failure (splintering), indicating good attachment to the porous wood surface.

#### Storage Stability (Shelf Life) of Two-Part System

A practical adhesive must remain stable over time during storage. The two-part system physically separates the reactive components (base generator vs polymerizable monomers) to enhance shelf life. Indeed, parts A and B individually showed excellent stability. After storing both parts for 6 months at room temperature, no visible changes were noted, and when mixed, they still cured normally with only a 10% increase in induction time (Figure 5).

**Figure 5:**
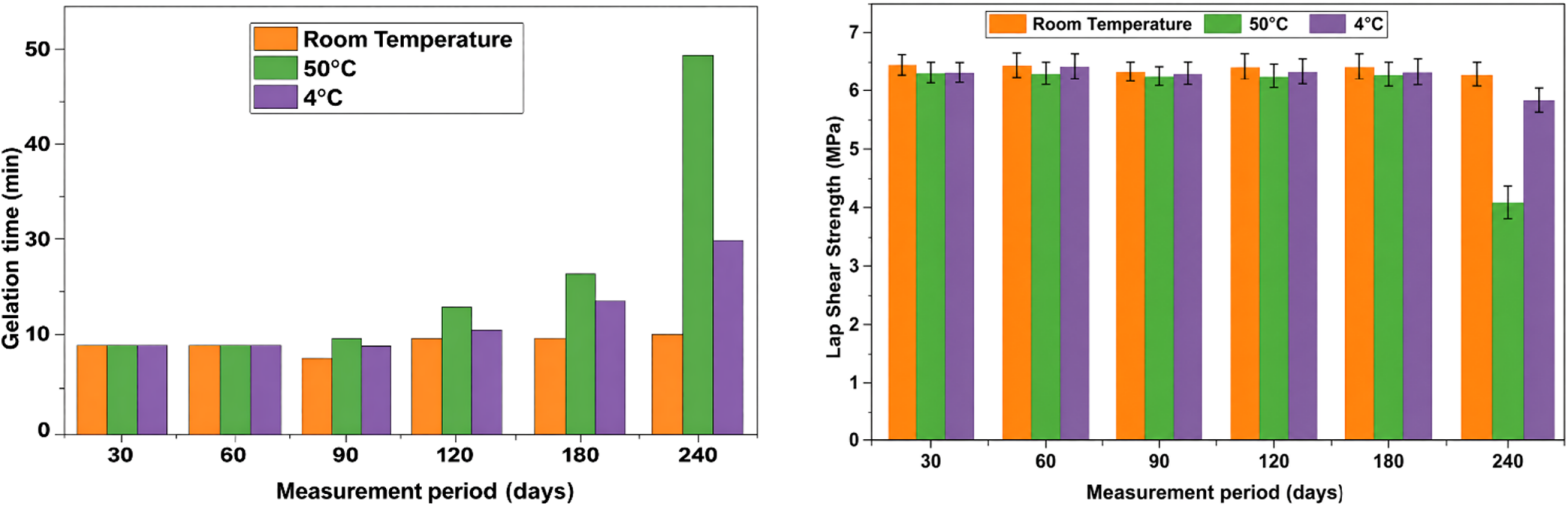
Effect of storage life on (left) gelation time and (right) lap shear strength of thiol-Michael addition-based time-lapse adhesives over time. Data was obtained using the optimized two-part adhesive formulation listed in Table 1 (TMPTMP/TMPTA with 7 wt % WMSP, 6 wt % acidic urea solution, and 9 wt % total fumed silica per part, mixed 1:1 by weight).

Accelerated aging tests were conducted by storing samples at 50 °C; some loss of activity was observed. After ∼4 months at 50 °C, the gelation time upon mixing increased substantially (to ∼50 min, versus ∼5 min when fresh), and the resulting polymer’s lap shear strength on PC dropped to ∼4 MPa (Figure 5). This degradation at high temperature can be attributed to the gradual deactivation of the enzyme because proteins can denature over time, and possibly slow side reactions (e.g., acrylate self-polymerization or thiol oxidation).^49–51^ In contrast, samples stored refrigerated at 4 °C maintained performance much better; even after 12 months at 4 °C, mixed samples gelled within ∼10 min and achieved ∼90% of their initial lap shear strength. Figure 5 summarizes these trends: at 50 °C storage, the gel time started to increase markedly after ∼200 days. The lap shear strength remained around 7 MPa for up to 6 months at 4 °C and room temperature, but for samples aged at 50 °C, it began dropping after ∼200 days. These results underscore the value of cold storage to maximize shelf life. Overall, the two-part adhesive demonstrated a shelf life well beyond 6 months at ambient or colder conditions.

### One-Pot Water-Activated Adhesive Performance

#### Formulation Advantages

The one-pot adhesive incorporated the same thiol–acrylate reactive system and enzymatic trigger but in a single package. The key distinction was that water was the activator rather than the mixing of two parts. This makes it particularly useful for scenarios in which applying an adhesive in wet conditions or curing underwater is required. Furthermore, eliminating the mixing step can reduce user error. Our optimized formulation (Table 1) included both hydrophilic and hydrophobic fumed silica in almost equal proportions (2.1% and 2.0%, respectively). This dual silica approach was found to be critical: the hydrophilic silica helped absorb and distribute the small amount of water and polar components (urea, PPA) in the system, while the hydrophobic silica ensured the bulk of the adhesive remained hydrophobic enough to resist premature curing from ambient humidity. The result was a stable paste that did not separate or flow, but allowed water penetration.

MEHQ and PPA additives significantly improved pot life. Without MEHQ and PPA, we observed that over a few weeks, some polymerization would occur. With MEHQ and PPA present, no noticeable curing or viscosity increase occurred over >2 months at room temperature, confirming that radical-mediated acrylate polymerization was effectively suppressed. PPA protonates the thiolate anions, keeping them as thiol (R–SH) and thus preventing any base-catalyzed Michael addition.^21^ We confirmed that a sealed sample stored for 8 weeks still had the same curing behavior (gel time ∼6 min after water addition) as a freshly made sample, demonstrating a pot life of at least two months.

#### Water-Triggered Curing and Underwater Bonding

When the one-pot adhesive was brought into contact with water, gelation was initiated within several minutes. A small amount of water (5–10% of the adhesive mass) was sufficient to dissolve the urea and PPA, creating a local aqueous phase that activated the WMSP and urease. For instance, extruding a bead of adhesive into a Petri dish and adding a few drops of water caused the bead to begin gelling in ∼3 min and fully solidify in ∼10 min (comparable to the two-part system’s timing). Notably, curing occurred even when fully submerged underwater. We demonstrated the bonding of glass and plastic substrates underwater by applying the adhesive to the surfaces and assembling them underwater. The adhesive cured and bonded the substrates within ∼15 min.

In lap shear tests performed on polycarbonate coupons that were bonded and cured entirely under water, the one-pot adhesive achieved a shear strength of ∼6 MPa. This was somewhat lower than the strength achieved in dry conditions for the two-part system on PC (∼7 MPa). The slightly reduced strength may be the result of the one-pot formulation having a slightly lower proportion of polymerizable monomers (77% total monomers vs 85% in the two-part) due to the inclusion of additives. A value of 6 MPa is significantly lower than the 12 MPa reported by Du et al.^52^ or the >10 MPa reported by Cheng et al.^53^ However, it is higher than some reports (1.2 MPa)^54^ and 3.9 MPa.^55^

An additional benefit of the water-activated cure was observed in gap-filling scenarios. The production of ammonia within the adhesive rather than from the environment means that even in submerged or confined joints, the cure propagates throughout the material. This is akin to certain moisture-curing adhesives, but here the mechanism is internal: as long as some water can penetrate or is mixed into the adhesive, the entire volume will eventually cure. The urea–urease reaction generates a basic front that propagates through the adhesive thickness, triggering polymerization uniformly.^21^

#### Adhesives Properties of One-Pot Formulation

We characterized the cured one-pot adhesive (activated and cured in air and under water) to compare it with the two-part system. The lap shear strength on PC (dry bonding with water activation) reached ∼6 MPa, slightly below the two-part’s 7 MPa, likely due to the formulation differences noted. On aluminum, the strength was again lower (∼3 MPa) with adhesive failure.

One interesting finding was that the one-pot adhesive showed improved bonding on wet surfaces (as intended): for example, bonding two pieces of wet wood (dipped in water and then bonded) resulted in a strength of ∼4 MPa. This highlights the potential of the system for use in underwater applications.

#### Hydrolytic Degradation Behavior (Gravimetric Analysis)

Because the one-pot adhesive is intended for underwater use, we examined its long-term stability in aqueous environments. Although the cross-linked thiol–acrylate network is hydrophobic, it does contain ester linkages that could be susceptible to hydrolysis. In a 3 month immersion test at room temperature, the cured adhesive samples gradually lost mass and strength. Over the first month, it is clear from Figure 6 that weight loss was modest (∼5%), and lap shear strength remained above 80% of the initial value. By 2 months (60 days), weight loss reached ∼15%, and lap shear strength dropped by ∼50%. The degradation accelerated thereafter, with samples at 3 months showing >25% mass loss and a loss of structural integrity. This behavior is consistent with a hydrolytic chain scission mechanism; likely the ester bonds in TMPTA or the mercaptopropionate are cleaving in the presence of water.

**Figure 6:**
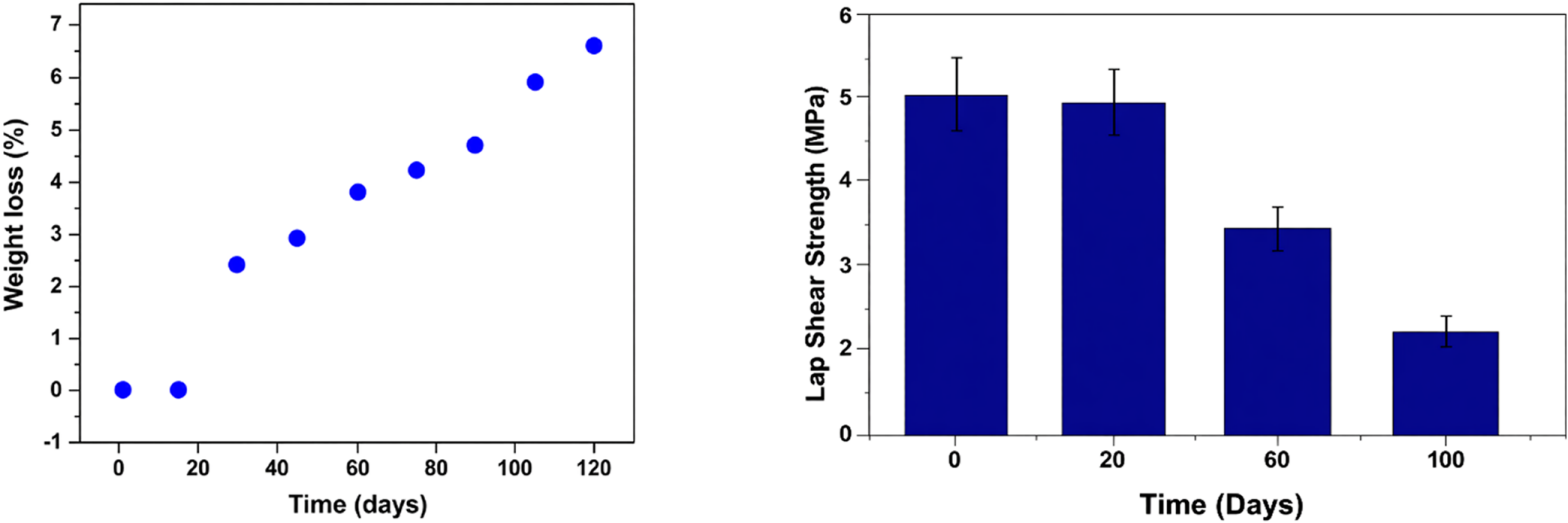
Degradation study of the water-activated, time-lapse adhesive for underwater applications, showing (left) weight loss (%) over time and (right) the corresponding decrease in lap shear strength (MPa) with degradation duration.

While this degradation is a drawback for permanent underwater applications, it could be advantageous for temporary adhesion needs. For now, the one-pot adhesive is best suited for scenarios where the bonded joint does not need to sustain high loads beyond a few weeks in continuously wet conditions or where periodic reapplication is feasible. It is noteworthy that in normal dry service, the adhesive showed no such degradation; samples aged at ambient humidity for months had no loss of strength, underscoring that the mechanism is specifically water-driven hydrolysis.

#### Impact of Filler Composition on Gelation Kinetics and Underwater Adhesive Strength

The influence of hydrophobic-to-hydrophilic fumed silica ratios on gelation behavior and underwater adhesive performance was systematically investigated. As shown in Figure 7, increasing the proportion of hydrophobic fillers prolongs the gelation time due to reduced water permeability, which delays ammonia generation and subsequently slows the base-catalyzed thiol-Michael reaction. Formulations with higher hydrophobic content demonstrated improved lap shear strength after 20 days of underwater exposure, attributed to the formation of a more water-resistant interfacial barrier. Notably, the formulation containing only hydrophobic filler exhibited rapid gelation failure, which refers to premature surface solidification without uniform bulk curing, caused by limited water permeability in fully hydrophobic filler formulations, which prevents effective urea dissolution and urease activation, underscoring the necessity of some hydrophilic content to facilitate water-triggered polymerization. These results highlight the importance of achieving an optimal hydrophobic/hydrophilic balance to tailor both curing kinetics and mechanical durability in aqueous environments. The formulation containing only hydrophobic filler exhibited rapid gelation failure, underscoring the necessity of some hydrophilic content to facilitate water-triggered polymerization.

**Figure 7:**
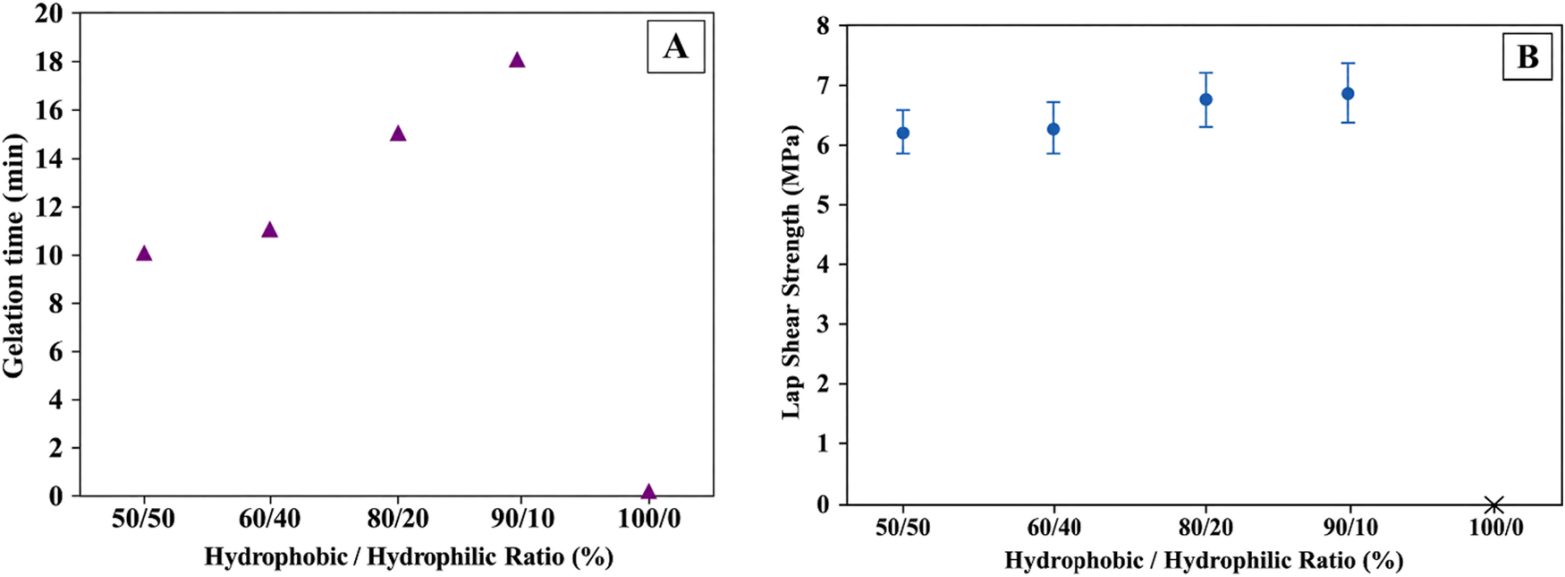
Effect of the variations in amounts of hydrophobic and hydrophilic silica on: (A) gelation time and (B) lap shear strength after 20 days of underwater exposure.

#### Tunable Gelation and Adhesion Properties in Thiol–Acrylate Time-Lapse Systems

In thiol–acrylate/urea–urease time-lapse adhesives, variation in WMSP content, urea concentration, and initial pH enabled tuning of gelation behavior and final adhesive strength. Below is a concise summary of how each parameter modulated performance, accompanied by representative plots.

#### Watermelon Seed Powder Concentration

Effect on gelation time: Increasing WMSP from 7 wt % to 15 wt % increased the rate of ammonia release, shortening the gelation time from ∼420 s to ∼180 s (Figure 8A).

**Figure 8:**
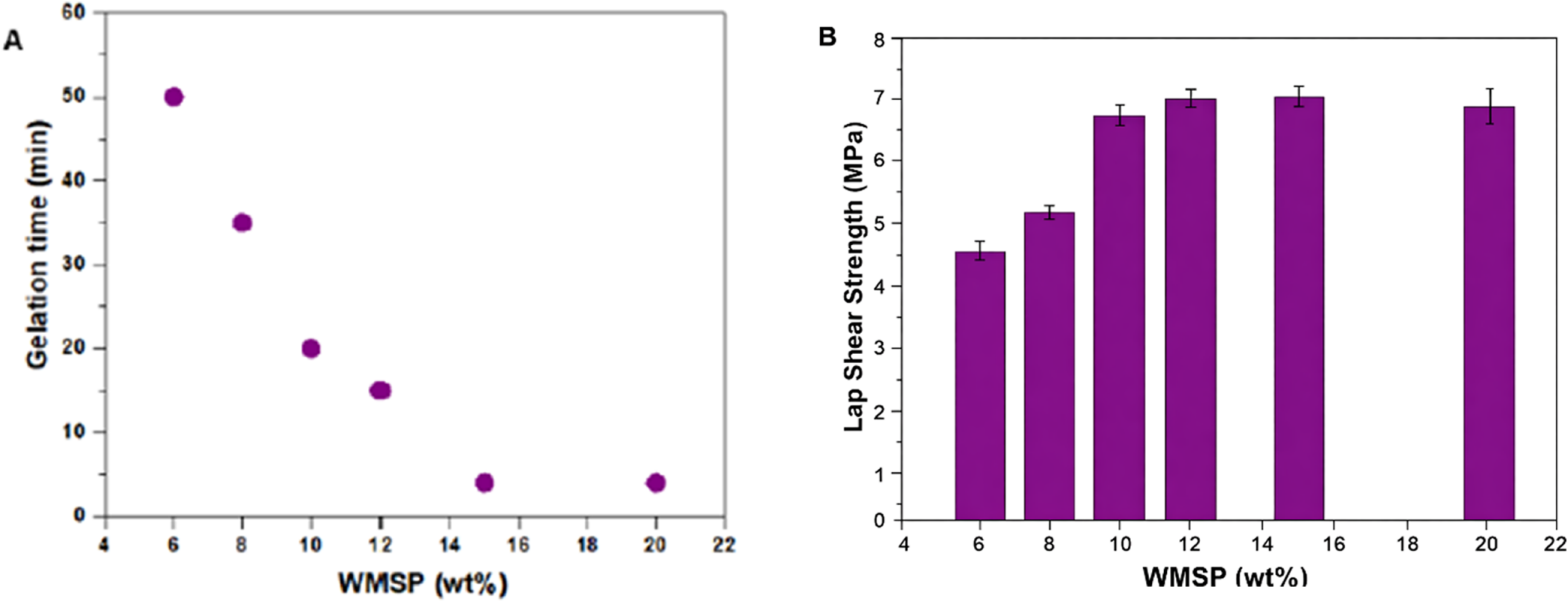
Effect of WMSP concentration on (A) gelation time and (B) lap shear strength of thiol-Michael addition-based time-lapse adhesives.

Effect on lap shear strength: Figure 8B demonstrates that lap shear strength rose from ∼2 MPa to ∼6 MPa, reflecting denser cross-linking and improved network cohesion with higher base-generator loading.

#### Urea Loading

Effect on gelation time: Elevating the urea concentration from 3 wt % to 9 wt % reduced the induction period, decreasing the gelation time from ∼480 s to ∼240 s as more substrate increased the final pH. (Figure 9A).

**Figure 9:**
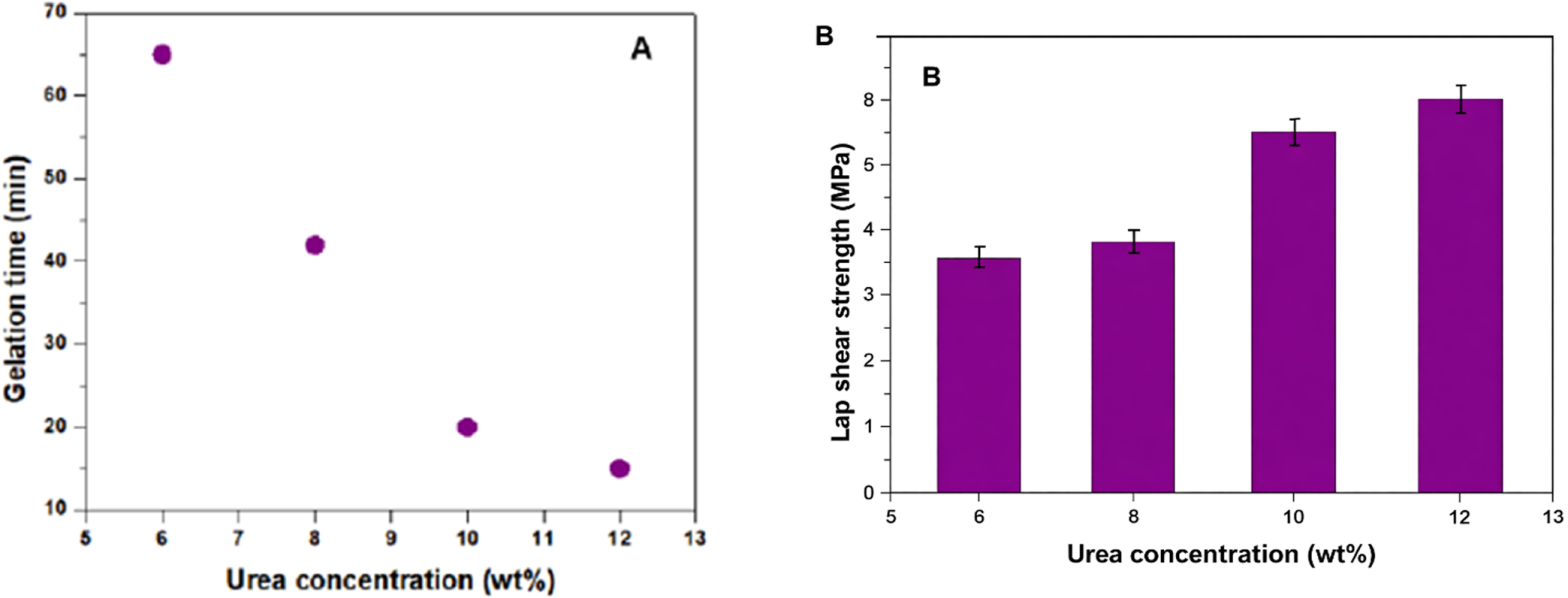
Effect of urea concentration on (A) gelation time and (B) lap shear strength of thiol-Michael addition-based time-lapse adhesives.

Effect on lap shear strength: The lap shear strength increased with an increase in urea concentrations, with a maximum of 5.5 MPa. (Figure 9b).

#### Initial Formulation pH

Effect on gelation time: A starting pH in the range of 4.0–5.5 delayed urease activation; raising the initial pH from 4.0 to 6.0 shortened the gelation time from ∼600 s to ∼150 s by bringing the system closer to the enzyme’s optimal activity window (Figure 10A).

**Figure 10:**
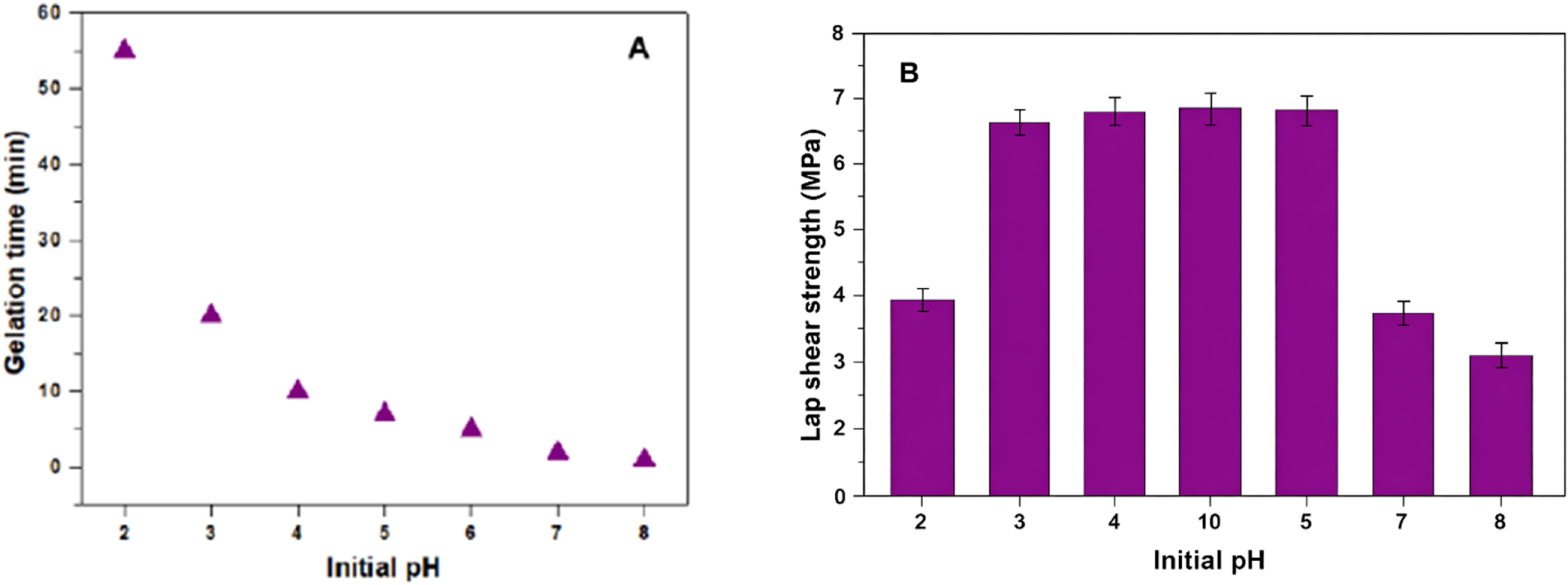
Effect of Initial pH on gelation time (A) and lap shear strength (B) of thiol–acrylate adhesives formulated with a urea–urease clock reaction.

Effect on lap shear strength: A near-neutral initial pH (∼6.5) yielded maximum adhesive strength (∼6.2 MPa), whereas overly acidic (pH < 4.5) or alkaline (pH > 7.5) conditions led to lower lap shear strength (Figure 10B).

## Conclusion

We have demonstrated two adhesive systems that leverage an enzymatic pH-clock reaction (urea–urease) to decouple the mixing and curing events, enabling time-lapse adhesion. The two-part thiol–acrylate adhesive used urease (from watermelon seeds) and urea to induce a delayed gelation, offering a tunable working time and strong final bond (up to ∼7 MPa) on various substrates. The one-pot water-activated adhesive built on the same chemistry, incorporating inhibitors and buffers to remain shelf-stable as a single component, demonstrated the ability to bond substrates underwater if the formulation was applied before immersion. The enzymatically generated ammonia successfully catalyzed thiol–acrylate polymerization in a controllable fashion, and the resulting polymer networks exhibit high strength.

Shelf life studies showed that the two-part system can be stored for months (especially under refrigeration) with minimal loss in reactivity, while the one-pot system, thanks to MEHQ and PPA, also achieved a pot life of at least 2 months. We also determined that one-pot samples did not react for at least 15 days when exposed to ambient humidity. The one-pot adhesive, when submerged in water, cured uniformly and adhered well, although extended water exposure (>2 months) gradually degraded the material.

These time-lapse adhesive systems open new possibilities in scenarios requiring delayed action or on-demand curing. For instance, in biomedical applications, a surgical adhesive or injectable filler could be designed to solidify only after a certain time postapplication, improving handling and placement before setting. Because the urea–urease reagents are biocompatible (urea and urease are nontoxic in the amounts used), the adhesive could be suitable for in vivo use, with the added benefit that the cured material can be made to degrade over time if desired (as shown by our hydrolytic degradation results).

The underwater formulation suggests utility in marine or plumbing repairs where other adhesives fail to cure. Moving forward, future work will focus on enhancing certain aspects: improving adhesion to metals and increasing the long-term durability of the underwater adhesive. Nonetheless, the results herein firmly establish the concept of cure-on-demand thiol–acrylate adhesives via an enzymatic clock reaction and illustrate their versatility in both two-component and one-component formats.
